# CRISPR/Cas9-Induced Inactivation of the Autism-Risk Gene *setd5* Leads to Social Impairments in Zebrafish

**DOI:** 10.3390/ijms24010167

**Published:** 2022-12-22

**Authors:** Chiara Gabellini, Cecilia Pucci, Chiara De Cesari, Davide Martini, Caterina Di Lauro, Matteo Digregorio, William Norton, Alessio Zippo, Alessandro Sessa, Vania Broccoli, Massimiliano Andreazzoli

**Affiliations:** 1 Unit of Cell and Developmental Biology, Department of Biology, University of Pisa, 56123 Pisa, Italy; 2 Scuola Superiore Sant’Anna, 56127 Pisa, Italy; 3 Department of Genetics and Genome Biology, University of Leicester, Leicester LE1 7RH, UK; 4 Laboratory of Chromatin Biology & Epigenetics, Center for Integrative Biology (CIBIO), University of Trento, 38123 Trento, Italy; 5 Stem Cell and Neurogenesis Unit, Division of Neuroscience, San Raffaele Scientific Institute, 20132 Milan, Italy; 6 CNR Institute of Neuroscience, 20132 Milan, Italy

**Keywords:** *setd5*, zebrafish, autism, neurodevelopment, behavior

## Abstract

Haploinsufficiency of the *SETD5* gene, encoding a SET domain-containing histone methyltransferase, has been identified as a cause of intellectual disability and Autism Spectrum Disorder (ASD). Recently, the zebrafish has emerged as a valuable model to study neurodevelopmental disorders because of its genetic tractability, robust behavioral traits and amenability to high-throughput drug screening. To model human *SETD5* haploinsufficiency, we generated zebrafish *setd5* mutants using the CRISPR/Cas9 technology and characterized their morphological, behavioral and molecular phenotypes. According to our observation that *setd5* is expressed in adult zebrafish brain, including those areas controlling social behavior, we found that *setd5* heterozygous mutants exhibit defective aggregation and coordination abilities required for shoaling interactions, as well as indifference to social stimuli. Interestingly, impairment in social interest is rescued by risperidone, an antipsychotic drug used to treat behavioral traits in ASD individuals. The molecular analysis underscored the downregulation of genes encoding proteins involved in the synaptic structure and function in the adult brain, thus suggesting that brain hypo-connectivity could be responsible for the social impairments of *setd5* mutant fishes. The zebrafish *setd5* mutants display ASD-like features and are a promising *setd5* haploinsufficiency model for drug screening aimed at reversing the behavioral phenotypes.

## 1. Introduction

Autism spectrum disorders (ASD) are a heterogeneous group of complex neurodevelopmental syndromes affecting approximately 1% of the human population. Typical symptoms of ASD include early-onset impairments in communication, learning and social interactions, as well as restricted interests and repetitive behaviors [1]. These manifestations can be associated with a variety of other symptoms, including intellectual disability (ID), hyperactivity, motor deficits and developmental delay. Although environmental factors can be important contributing factors, recent studies have accumulated a large body of evidence indicating that mutations in an increasing number of genes can cause ASD. Most of the ASD-risk genes code for proteins involved in transcription, protein synthesis and degradation, as well as neurogenesis and synaptogenesis [2,3]. The first category is of particular interest and includes genes that code for transcription factors and chromatin remodelers, which are expected to act at a higher hierarchical level than the other classes of genes, being able to regulate the expression of target genes and thus triggering the activation of specific genetic programs. Furthermore, chromatin regulators are known to play key roles in various aspects of neural development, including progenitor specification, cell-type specific differentiation, migration and generation of mature neural networks [4]. As a consequence, the impairment of chromatin remodeling results in crucial deficiencies in circuit formation and cognitive functions. Recently, converging evidence has indicated that heterozygous loss of function (LoF) mutations in the gene *SETD5*, encoding a SET domain-containing histone-modifying protein, are one of the most frequent genetic causes of both ID and ASD [5,6,7,8,9,10,11,12,13]. Several studies focusing on the molecular function of *SETD5* have highlighted distinct, non-mutually exclusive, chromatin-regulating activities, involving this protein in histone methylation [14,15] as well as in the interaction with the polymerase-associated factor 1 (PAF1) complex and histone deacetylase 3 (HDAC3) complex [15,16,17,18,19]. Functional studies performed in mice indicated that *Setd5* haploinsufficiency negatively affects the expression of neurodevelopmental genes, in particular those associated with synaptic functioning, learning and memory, eventually leading to an ASD-like phenotype, which includes cortical hypoconnectivity, cognitive deficit, altered social interactions and impairments in adaptive behavior [15,20].

Recently, the zebrafish (*Danio rerio*) has been recognized by SFARI, one of the major foundations supporting autism research, as a rapidly emerging model to study ASD [21]. Indeed, the zebrafish model offers a variety of advantages including a large number of offspring, rapid external development of its transparent embryos, the availability of classical genetic as well as reverse genetic approaches and a genome sharing over 70% of its genes with humans [22]. Furthermore, zebrafish and mammals display strong similarities in neural cell types as well as conserved signaling pathways. Finally, the availability of specific tests to assess different aspects of the complex behaviors of this social fish, makes zebrafish a powerful complementary model system to mouse, to elucidate the function of ASD risk genes and perform high-throughput drug screening.

In the present work, we used the CRISPR/Cas9 technology to generate the first zebrafish *setd5* mutant line. As human patients are characterized by *SETD5* haploinsufficiency, we focused our analysis on zebrafish heterozygous *setd5* mutants. Interestingly, we found that these mutants display ASD-like features and therefore they can be proposed as novel system to model and study key aspects of *SETD5* haploinsufficiency in humans.

## 2. Results

### 2.1. setd5 Is Expressed in Zebrafish Adult Brain and during Early Embryo Development

**Figure 1:**
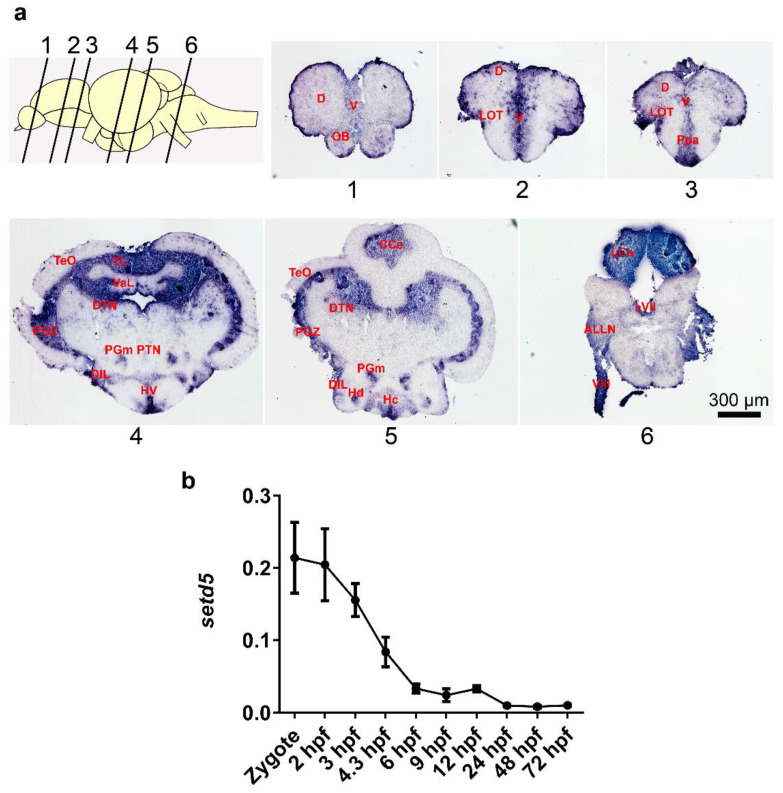
*setd5* is expressed in the zebrafish adult brain and at early stages of zebrafish embryo development. (**a**) *setd5*-203 isoform localization on zebrafish WT brain sections at 10/12 months of age performing in situ hybridization technique. Representative images of a section corresponding to telencephalon (1–3), diencephalon-mesencephalon (4,5) and rhombencephalon (6), *n* = 3 brains analyzed. Scale bar 300 µm. (**b**) Expression of *setd5* isoforms in zebrafish embryos and larvae at different developmental stages, obtained by RT-qPCR analysis. Data are expressed as 2^−(ΔCt)^ mean ± Standard Error of the Mean (SEM), using *b-actin1* as housekeeping gene. *n =* 3 independent experiments. Abbreviations: D, Dorsal telencephalic area; V, Ventral telencephalic area; OB, Olfactory bulb; LOT, Lateral olfactory tract; Ppa, Parvocellular preoptic nucleus; TL, Longitudinal Torus; Val, Lateral division of valvular cerebelli; TeO, Tectum opticum; PGZ, Periventricular gray zone of optic tectum; DTN, Dorsal tegmental nucleus; HV, Ventral zone of periventricular hypothalamus; PTN, Posterior tuberal nucleus; PGm, Medial progromerular nucleus; DIL, Diffuse nucleus of the inferior lobe; Hc, Caudal zone of periventricular hypothalamus; Hd, Dorsal zone of the periventricular hypothalamus; CCe, Cerebellar corpus; LCa, Caudal lobe of cerebellum; LVII, Facial lobe; ALLN, Anterior lateral line nerves; VIII, Octaval nerve; hpf, hours post fertilization.

In order to characterize the localization of *setd5* mRNA in zebrafish central nervous system (CNS), we analyzed the expression of *setd5* transcript on WT brain sections of adult individuals (10/12 months of age) by in situ hybridization. As shown in Figure 1a, *setd5* is expressed in different areas of the telencephalon (Figure 1a, slices 1–3) (including telencephalic dorsal area, telencephalic ventral area, olfactory bulb and lateral olfactory tract), diencephalon (Figure 1a, slices 4,5) (including parvocellular preoptic nucleus, ventral hypothalamus, posterior tuberal nucleus, medial preglomerular nucleus, diffuse nucleus of the hypothalamus lower lobe, caudal area of the periventricular hypothalamus and dorsal zone of the periventricular hypothalamus), mesencephalon (Figure 1a, slices 4,5) (including longitudinal torus, optical roof, periventricular gray area of the optical roof and dorsal nucleus of the tegmentum) and of the rhombencephalon (including the cerebellar valvula, cerebellar body, cerebellar caudal lobe, LVII facial lobe and nerves of the anterior lateral line) and in the eighth cranial nerve (Figure 1a, slice 6).

We also analyzed the expression levels of the *setd5* transcript during zebrafish embryo development from the zygote stage up to 72 h post-fertilization (hpf), observing relatively high expression levels already observed at zygote stage (Figure 1b). This early appearance of *setd5* RNA, also previously reported by whole mount in situ hybridization experiments [15], indicate the presence of maternal transcripts. This evidence suggests an early role played by the Setd5 protein during the first stages of embryo development.

### 2.2. setd5 Knock-Out Causes a Growth Delay in Zebrafish

**Figure 2:**
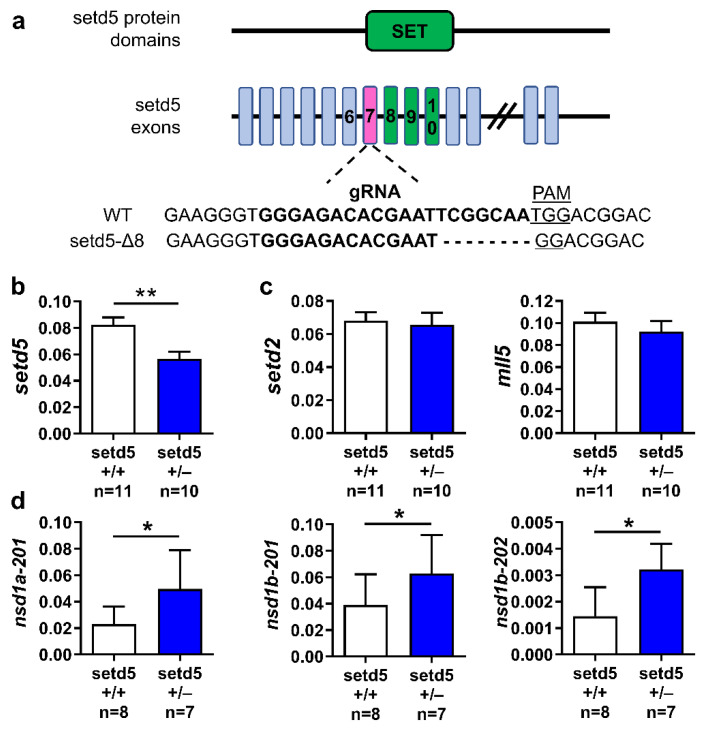
Location of the eight-nucleotide deletion in *setd5* gene (setd5-Δ8) and its effects on the expression of selected genes. (**a**) Schematic representation of the main zebrafish Setd5 protein domains and the corresponding exons, highlighting the SET domain (in green). The 20-nucleotide sequence corresponding to gRNA in exon 7 (in magenta) encoding sequence is indicated, as well as the PAM sequence, aligned to the deleted *setd5* mutant sequence obtained by CRISPR/Cas9 gene editing technique. (**b**–**d**) Expression of *setd5* (**b**), *setd5* paralogues *setd2* and *mll5* (**c**), *nsd1a* and *nsd1b* isoforms (**d**) in *setd5*+/+ and *setd5*+/− adult zebrafish brains, obtained by RT-qPCR analysis. The values are expressed as 2^−(ΔCt)^, using *bactin1* as the housekeeping gene. (**b**–**d**) *n =* number of adult brains analyzed. Data are expressed as mean ± SEM. Statistical analysis was performed by Student’s *t*-test. * *p* < 0.05; ** *p* < 0.01.

Since we previously demonstrated that *setd5* knock-down affects zebrafish embryonic development [15], we took advantage of the CRISPR/Cas9 genome-editing technique to generate a stable mutated zebrafish model for the *setd5* gene. Cas9 mRNA was injected into the zygote cell along with a guide RNA, targeting the *setd5* exon 7 which is upstream of those encoding the protein functional domain SET (Figure 2a). A representative group of injected F0 embryos were processed by Melting analysis to confirm the gene editing event (Figure S1 (Supplementary Materials)). Once they reached the adult stage, the mosaic founders able to transmit mutations to their progeny were identified by Melting analysis on heterozygous F1 generation embryos obtained after outcrossing with WT individuals. Many indel mutations were identified and we focused on a deletion of eight nucleotides: this frameshift mutation resulted in a putative LoF of *setd5* gene since it determines the appearance of a premature stop codon (Figure S2). We then confirmed the reduced expression of *setd5* transcript in *setd5* mutant adult brains (Figure 2b), which may lead to the activation of compensatory mechanisms of gene expression, a well-demonstrated phenomenon in zebrafish [23]. Indeed, while the expression of the *setd5* paralogues *setd2* and *mll5* in the adult brain was not different between *setd5* mutants and WT ones (Figure 2c), heterozygous mutants showed a significant increase in expression of nsd1a and nsd1b (both the isoforms), the orthologues of human NSD1 (Figure 2d), encoding a histone methyltransferase that shares the same amino acidic target, Lysine 36 of histone 3, with *SETD5* [24]. 

**Figure 3:**
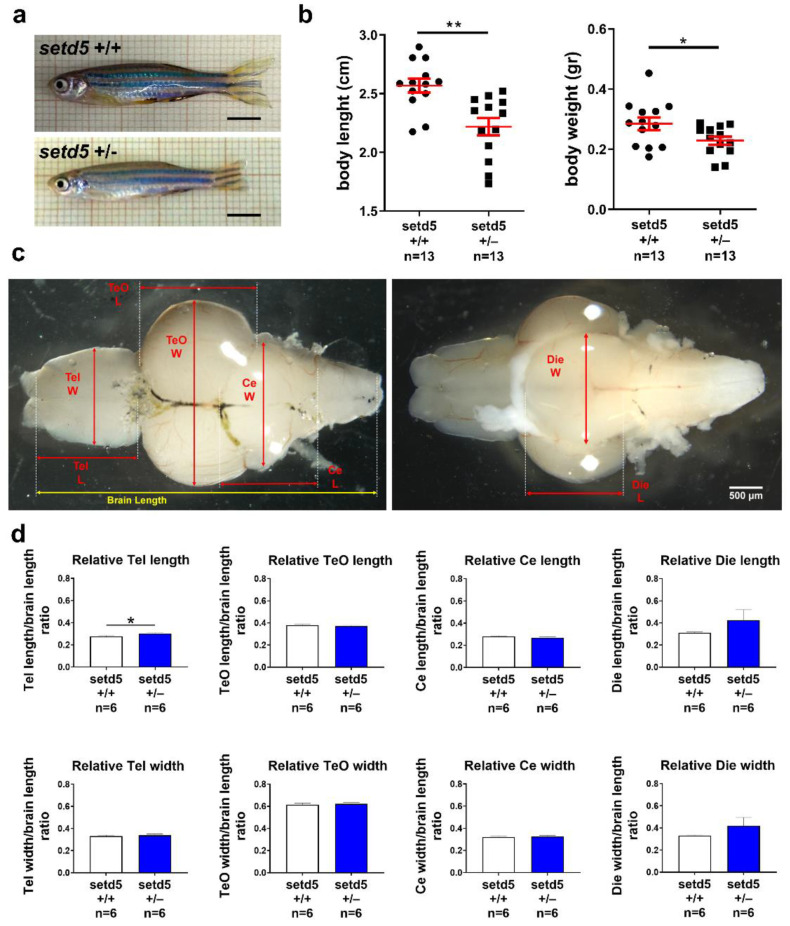
*setd5* haploinsufficiency causes a growth delay in adult mutant zebrafish. (**a**) Representative images of 10-month-old *setd5*+/+ and *setd5*+/− zebrafish adults. Scale bar 500 μm. (**b**) Body length and body weight analysis in *setd5*+/+ and *setd5*+/− 10 months-old zebrafish adults. *n =* number of adults analyzed. (**c**) Representative images of adult brains from 10-month-old *setd5*+/+ zebrafish adults, evidencing the different measurements conduced for the morphometrical analysis included in (**d**). W, width; L, length. (**d**) Relative Telencephalon (Tel) length and width, Optic tectum (TeO) length and width, Cerebellum (Ce) length and width, Diencephalon (Die) length and width in adult brain, normalized to the entire brain length, from 10-month-old *setd5*+/+ zebrafish adults. Absolute values are included in Figure S5. (**d**) *n =* number of adult brains analyzed. Data are expressed as mean ± SEM. Statistical analysis was performed by Student’s *t*-test. * *p* < 0.05; ** *p* < 0.01.

As shown in Figure 3a,b, body length and body weight were significantly reduced in mutant *setd5*+/− zebrafish adults when compared to *setd5*+/+ individuals. A morphometric analysis of explanted brains indicated that in mutant fishes the length of the telencephalon normalized to the length of the entire brain is significantly increased compared to wild type fishes (Figure 3c,d). As the volume of the entire brain is not different between mutants and wild type fishes (Figure S4), these data suggest that setd5+/− fishes display an alteration of the relative morphological proportions of the telencephalon compared to other brain districts. 

### 2.3. setd5 Mutant Embryos Show Growth Delay, Microphthalmia and Deficits in Locomotor Behavior

**Figure 4:**
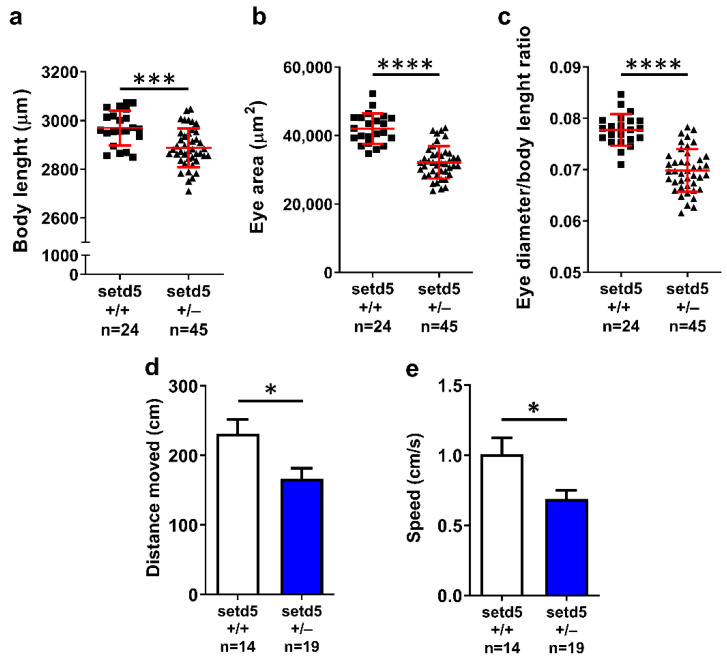
*setd5* haploinsufficiency impairs the growth in mutant zebrafish embryos. (**a**) Body length, (**b**) eye area and (**c**) eye diameter/body length ratio analysis in *setd5*+/+ and *setd5*+/− embryos at 2 days post fertilization (dpf). (**d**) Distance moved and (**e**) speed of *setd5*+/+ and *setd5*+/− larvae at 6 dpf evaluated during a 5-min-long open field test. (**a**–**e**) Statistical analysis was performed using the Student’s *t*-test. Data are expressed as mean ± SEM; *n =* number larvae analyzed. * *p* < 0.05; *** *p* < 0.001; **** *p* < 0.0001.

We also performed morphometric analysis on *setd5* mutant embryos at 2 days post-fertilization, to check if the altered phenotype observed in knock-down embryos, characterized by microcephaly, reduced eye size, cerebral and pericardial edema [15], could also be observed in stable *setd5* mutants. Mutant embryos showed a reduced body length (Figure 4a) and eye area (Figure 4b) compared to *setd5*+/+ ones, although neither cerebral nor pericardial edemas were detected in mutant embryos. In addition, the ratio between the eye diameter and the body length was decreased in mutant embryos compared to *setd5*+/+ ones (Figure 4c), which is indicative of microphthalmia. To look for potential microcephaly during an early phase of brain development, we analyzed the brains of 24 hpf embryos. However, we did not find significant differences between *setd5*+/- and *setd5*+/+ embryonic brain areas (Figure S4a). Furthermore, in the same brains no difference was detected in the level of phospho-histone H3 and HuC/D used as markers of proliferation and neuronal differentiation, respectively (Figure S4b,c). 

**Figure 5:**
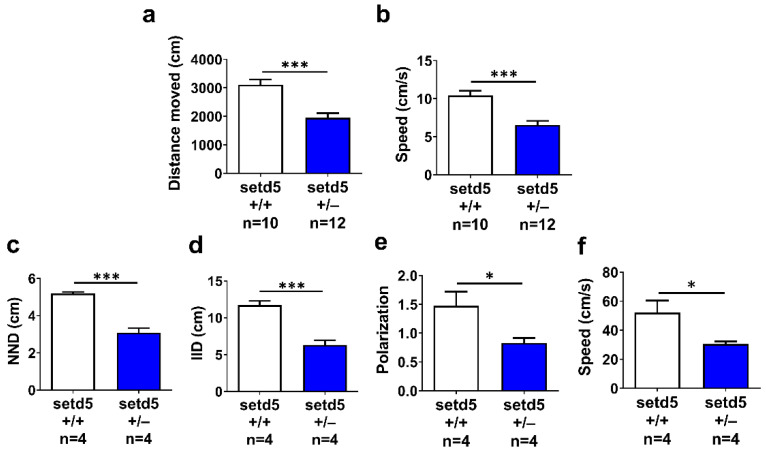
*setd5* haploinsufficiency alters locomotor activity and induces an anxiety-like shoaling behavior in adult mutant zebrafish. (**a**) Distance moved and (**b**) speed (cm/s) of *setd5*+/+ and *setd5*+/− adult fish evaluated during a 5-min-long open field test.; *n =* number of fishes analyzed. (**c**) Nearest Neighbor Distance (NND), (**d**) Inter-Individual Distance (IID), (**e**) polarization and (**f**) mean speed of fish groups constituted by 4 *setd5*+/+ or *setd5*+/− adults, evaluated by a shoaling test. (**a**–**f**) *n =* number of group of fish analyzed. Data are expressed as mean ± SEM. Statistical analysis was performed by Student’s *t*-test. * *p* < 0.05; *** *p* < 0.001.

We tested the locomotor activity of *setd5*+/− larvae at 6 dpf and observed a decrease in total distance swum (Figure 4d) and speed (Figure 4e) in *setd5*+/− larvae compared to *setd5*+/+ ones and a similar result was obtained for *setd5* knock-down embryos. To extend our analysis of locomotor behavior to *setd5*+/− fishes at the adult stage, we performed an open field test to evaluate the ability of the fish to explore a novel environment. As observed in *setd5*+/− larvae, adult *setd5*+/− fish showed a significant decrease in total distance swum (Figure 5a) associated with reduced speed (Figure 5b), compared to *setd5*+/+ counterparts.

### 2.4. setd5 Mutant Adults Show a Tight Shoal

Zebrafish exhibit shoaling behavior, the complex interaction of animals moving together in coordinated movements with polarized direction. Shoal cohesion is usually stable and maintains a relatively high baseline level in adult zebrafish. The shoaling test was used to assess overall social behaviors in groups of four individuals. Compared to WT, *setd5*+/− adults tended to swim closer together, in tighter shoals as demonstrated by the reduced Nearest Neighbor Distance (NND; Figure 5c) and a decreased Inter-Individual Distance (IID; Figure 5d). Moreover, *setd5*+/− shoals presented a disrupted polarization compared to *setd5*+/+ fish group (Figure 5e) and swam with a reduced speed (Figure 5f), demonstrating an alteration of social behavior generally associated to an anxiety condition.

### 2.5. setd5 Mutant Adults Display Perturbed Social Interaction, Ameliorated by the Antipsychotic Drug Risperidone

**Figure 6:**
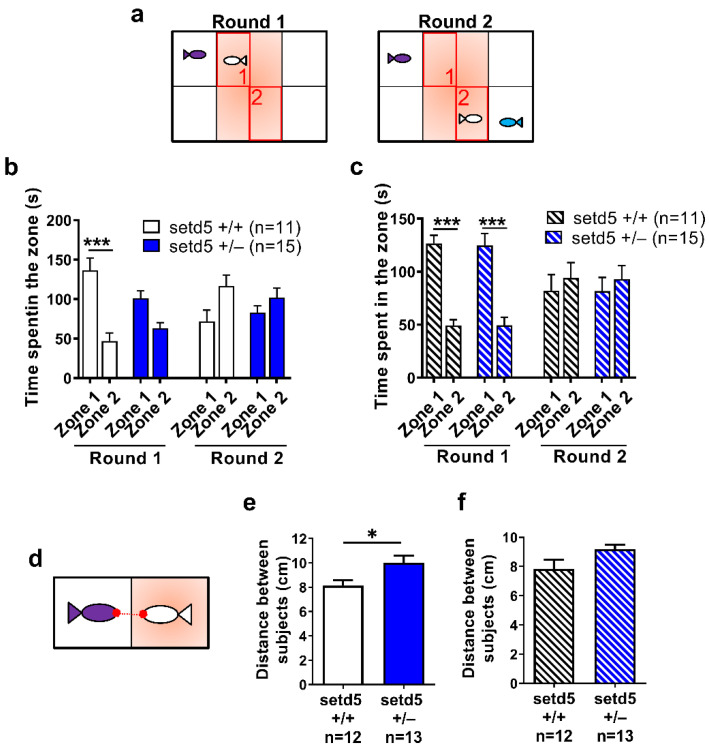
The antipsychotic drug Risperidone ameliorates the social behavior impairment of *setd5* adult mutant zebrafish. (**a**) Set-up of zebrafish adult fish during the two rounds of social preference test on the fish (white) included in the central arena. Zone 1 and zone 2, highlighted in red, indicates the areas of the arena near to the first stimulus (purple) and social novelty stimulus (blue) added at round 2. (**b**,**c**) Time spent by *setd5*+/+ and *setd5*+/− adult fish in zone 1 and zone 2, in both rounds of social preference test, for untreated (**b**) and Risperidone-treated fishes (**c**). (**d**) Set-up of the social behavior test, evidencing the distance between the nose points (in red) between the tested fish (white) and the social stimulus (purple). (**e**,**f**) Social behavior of *setd5*+/+ and *setd5*+/− adult fish, evaluated in terms of distance between the nose points of the subjects, untreated (**e**) and Risperidone-treated fish (**f**). (**b**,**c**,**e**,**f**) Data are expressed as mean ± SEM; *n =* number of fish analyzed. (**b**,**c**,**e**,**f**) Statistical analysis was performed using (**b**,**c**) 1-way ANOVA followed by post-hoc test or (**e**,**f**) Student’s *t*-test. * *p* < 0.05; *** *p* < 0.001.

Sociality is an essential component of zebrafish behavior that is easily observable and further increases the value of this species in modelling brain disorders involving deficits in social behavior, including ASD [25]. We performed a social preference test (Figure 6a) to evaluate the reaction of adult zebrafish individuals to a social stimulus as well as to a social novelty [25]. As expected [26], during round 1, the *sedt5*+/+ fishes spent significantly more time close to the stimulus fish in zone 1, compared to zone 2 (empty zone) which is far from the social stimulus. On the contrary, there was no significant difference between the time spent in zone 1 and in zone 2 by *setd5* mutant fishes, indicating an impairment of social interest (Figure 6b). Once a novel social stimulus, represented by another fish, was added in round 2, the sedt5+/+ fishes started to spend an equal time in zone 2, which is close to the additional fish, and in zone 1 which is nearby the first social stimulus. In this second round, *setd5*+/− fish displayed the same behavior observed during round 1, spending the same amount of time in zone 1 and zone 2 (Figure 6b). We next repeated the social preference test exposing zebrafish sedt5+/+ and *setd5* mutant fishes to risperidone, an antipsychotic drug commonly used to treat behavioral traits in ASD patients. In the case of *sedt5*+/+ fishes, in round 1 we obtained similar results as those seen in the absence of the drug. Interestingly, in the *setd5* mutant fishes we observed a significant increase in the time spent in zone 1 compared to zone 2, indicating a rescue of the social interest by risperidone treatment. During round 2, we did not observe a significant difference between the time spent in zone 1 and zone 2, in either WT or *setd5*+/− fishes, as observed in the untreated fishes (Figure 6c).

To confirm that *setd5* haploinsufficiency affects the social interest of adult fishes, we performed a social interaction test, tracking the nose point, beyond center point and tail base, of the individuals (Figure 6d). As shown in Figure 6e, the *setd5* mutant zebrafish showed a significant increase in the distance between the nose point of the tested fish and the nose point of the stimulus, which was abolished by the treatment with risperidone (Figure 6f).

### 2.6. setd5 Mutation Affects the mRNA Expression Levels of Proteins Involved in Neurotransmission

**Figure 7:**
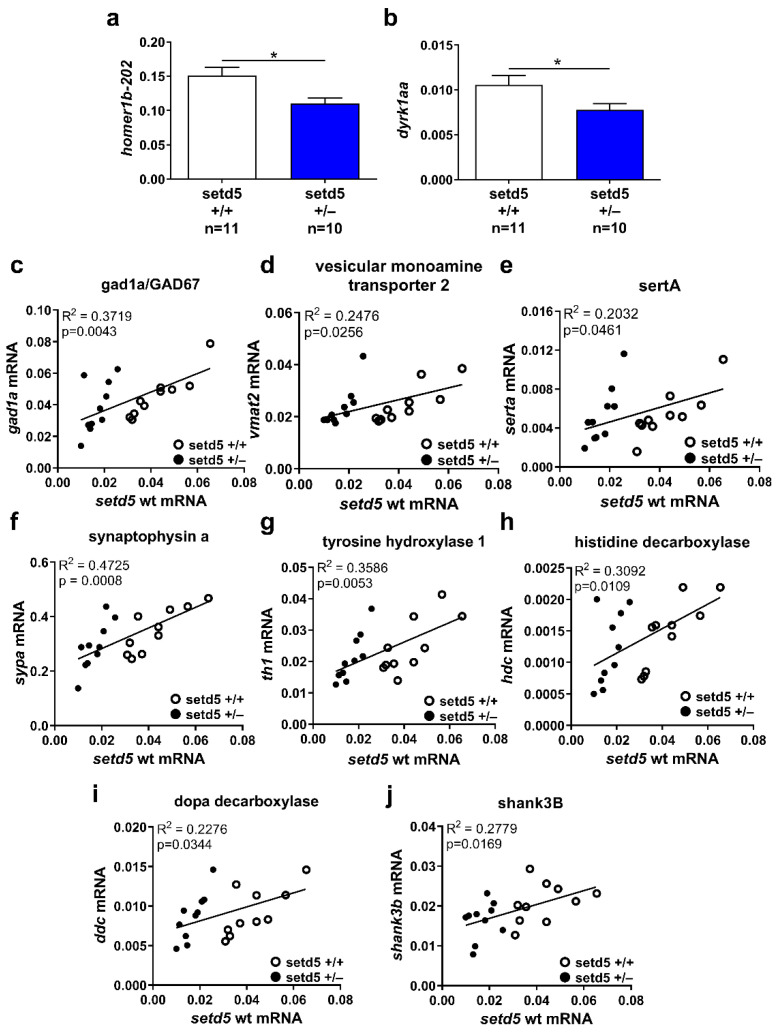
*setd5* haploinsufficiency affects mRNA expression levels of synaptic regulators. (**a**,**b**) Expression of *homer1b-202* (**a**) and *dyrk1aa* (**b**) in *setd5*+/+ and *setd5*+/− adult zebrafish brains, obtained by RT-qPCR analysis. The values are expressed as 2^−(ΔCt)^, using *bactin1* as the housekeeping gene. *n =* number of adult brains analyzed. Data are expressed as mean ± SEM. Statistical analysis was performed using a Student’s *t*-test. * *p* < 0.05. (**c**–**j**) Linear regression analysis between *setd5* wild type mRNA level and *gad1a/GAD67* (**c**), *vesicular monoamine transporter 2* (**d**), *sertA* (**e**), *synaptophysin a* (**f**), *tyrosine hydroxylase 1* (**g**), *histidine decarboxylase* (**h**), *dopa decarboxylase* (**i**), and *shank3B* (**j**) in *setd5*+/+ (empty dots) and *setd5*+/− (full dots) adult zebrafish brains, obtained by RT-qPCR analysis. R^2^ = coefficient of determination. A linear regression-fitting curve is shown.

Since neurotransmission is the basis of neuronal communication and is critical for normal brain development, behavior, learning and memory, we investigated different neurotransmitter pathways and proteins involved in neuronal activity in brain samples from adult *setd5* mutants. In particular, we evaluated the mRNA expression levels of *gad2*, *gad1a*, *gad1b* (GABAergic system), *dbh*, *dopa decarboxylase*, *dat*, *tyrosine hydroxylase 1* and *2* (catecholaminergic system), *tph1a*, *tph1b*, *tph2*, *serta* and *sertb* (serotoninergic system), *histidine decarboxylase 1* (histaminergic system), vesicular monoamine transporter *vmat2*, the differentiated neuronal marker *elavl3*, the transcripts encoding for the synaptic proteins *dyrk1aa* and *dyrk1ab*, *PSD95* and *synapsin 1*, *synaptophysin a*, *synaptophysin b*, *mecp2*, *nrxn1a*, *nrxn1b*, *shank3a* and *shank3b*. In particular, we investigated a possible relationship between the expression level of these transcripts and the levels of WT *setd5* mRNA, evaluated using specific primers that do not amplify mutant *setd5* transcript (Figure S5). Although a comparison between brain samples from *sedt5*+/+ and *setd5*+/− fishes did not show any significant difference in the expression level for most of the analyzed genes (Figure S6), we found that *setd5* mutant brains specifically express reduced mRNA levels of *homer1*, encoding a component of the post-synaptic complex [27] (Figure 7a) and *dyrk1aa* (Figure 7b), which encodes for a kinase involved in various cellular processes including pluripotency maintenance, synapsis function, neuronal differentiation and whose orthologue in humans is located in the Down Syndrome critical region (DSCR) on chromosome 21 [28]. Interestingly, there is a positive correspondence between WT *setd5* transcript levels and the expression of *gad1a* (Figure 7c), *vmat2* (Figure 7d), *sertA* (Figure 7e), synaptophysin a (Figure 7f), *tyrosine hydroxylase 1* (Figure 7g), histidine decarboxylase 1 (Figure 7h), *dopa decarboxylase* (Figure 7i) and *shank3B* (Figure 7j).

## 3. Discussion

In this study we have generated the first zebrafish *setd5* mutant line using CRISPR/Cas9 technology. We focused on heterozygous mutants that recapitulate human *SETD5* haploinsufficiency and characterized their morphological, molecular and behavioral phenotypes.

In zebrafish, *setd5* is expressed in the developing neural system since its initial specification [15] and its expression persists in many areas of the zebrafish adult brain including areas corresponding to ASD-affected regions such as the lateral zone of the dorsal telencephalic area, which is considered homologous to the mammalian hippocampus and preoptic area [29]. To model *SETD5* haploinsufficiency in humans, we generated *setd5* mutant zebrafish lines that carry specific deletions leading to the creation of a premature stop codon upstream of exons coding for the SET domain. The significant reduction in total *setd5* mRNA observed in the brain of heterozygous mutants compared to *sedt5*+/+ brain samples suggests that the mutant *setd5* transcripts may be subjected to nonsense-mediated decay, as previously demonstrated in a de novo *setd5* mutation isolated in a patient with early-onset epileptic encephalopathy [30]. Interestingly, gene expression analysis aimed at looking for potential compensatory mechanisms in the brains of *setd5* mutants did not detect differences in the expression level of the *setd5*-related genes *mll5* and *setd2*, while there was a significant upregulation of *nsd1a* and *nsd1b* expression. Interestingly, human NSD1 protein displays mono- and di-methylation activity on the H3K36 residue, which is in turn a target of *SETD5* leading to a three-methylated form of the amino acid. This might suggest that enhanced *nsd1a* and *nsd1b* expression may partially compensate for the effects of *setd5* haploinsufficiency in zebrafish heterozygous mutants.

In addition, we observed that *setd5* heterozygous embryos show a reduced body length compared to *setd5*+/+ ones, while the ratio between eye size and body length indicates that they would be affected by microphthalmia. This is very similar to the morphological phenotype described for zebrafish embryos injected with *setd5* morpholino and heterozygous mouse *Setd5* mutants [15,16,31]. It also represents a common clinical feature of individuals affected by human 3p25.3 microdeletion syndrome, in which one copy of *SETD5* is entirely deleted [9], further supporting the idea that the zebrafish *setd5* mutants carry a LoF mutation. Nevertheless, although the adult zebrafish mutants are characterized by a reduced body length and weight, they display a normal eye–head/total length ratio, suggesting a possible rescue of the microphthalmia phenotype which may be linked to the upregulation of *nsd1a* and *nsd1b* expression. We also found that, differently from embryos injected with *setd5* morpholino [15], zebrafish *setd5* mutants do not display microcephaly, neither at embryonic stages, nor during adulthood (Figures S3 and S4), although we noted an alteration of relative morphological proportions of the telencephalon compared to other brain districts. The differences between *setd5* knockdown embryos and *setd5*+/− embryos may reside in the fact that *setd5* morpholino can impair the translation of both maternal and zygotic *setd5* transcripts. Accordingly, the effects in *setd5* knockdown embryos are possibly stronger than those observed in *setd5* heterozygous mutants, which carry one wild type allele and a second allele in which the mutation more specifically affects the zygotic component of *setd5* mRNA. In future experiments, it will be interesting to address the role of the *setd5* maternal transcript through the generation of maternal-zygotic mutants.

The behavioral studies conducted in this work indicate that our zebrafish *setd5* mutant model may effectively replicate behavioral aspects typically altered in individuals heterozygous for *SETD5* mutations. An initial analysis in the open field test indicated that *setd5* heterozygous larvae and adults display a significant reduction in the distance moved and velocity when compared to *setd5*+/+ ones. This observation is in line with a significant deficit in motor abilities described for *SETD5* haploinsufficient individuals, although this phenotype has not been previously observed in *Setd5*+/− mice [9,13,15,16]. Similarly, previous studies have demonstrated that the zebrafish inactivation of other genes that play an important role in ASD and ID, such as *mecp2* [32] and *shank3b* [33], also results in reduced locomotor activity. In a shoaling test, aimed at evaluating the robust zebrafish behavior of aggregating and coordinately adapting to each other’s movements, we observed that *setd5* heterozygous adults exhibit tight shoaling, an altered behavior that is typically related to a condition of anxiety [34]. This is in keeping with the observation that ASD patients carrying *SETD5* mutations, as well as *Setd5*+/− mice, are characterized by increased levels of anxiety [13,20].

This behavior in the shoaling test is not typical of all fish models for ASD. For instance, *dyrk1aa* fish mutants display a loss of social cohesion [35]. However, mutations in other autism risk genes, such as *immp2l* and *adra1aa*, also lead to fish that exhibit tighter shoaling, indicating a convergent social phenotype, at least for a subset of zebrafish ASD models [36]. Furthermore, our data on *setd5* behavior, showing that heterozygous fish display a low swimming speed together with a tight shoaling and a low polarization, complement previous observations indicating that high speed correlates with large inter-individual spacing and group polarization [36]. Additional defects in social interactions were further uncovered by the social preference test. In particular, we found that *setd5*+/− adults appeared to be indifferent to a social stimulus, represented by a new fish introduced during the test. When compared to control fishes, this lack of social interest displayed by mutant fishes is shown by both the reduced time spent in the proximity of the stimulus and the increased distance kept between the tested fish and the stimulus. A similar phenotype is observed in *Setd5*+/− mice, as well as in other models of ASD, such as *dyrk1aa* zebrafish mutants [34] and Synapsin knockout mice [37], both of which display abnormalities in sociability. Interestingly, we found that the altered social behavior, observed for *setd5* zebrafish mutants in the social preference test, is significantly rescued following treatment with the antipsychotic drug risperidone, which is used to treat irritability in ASD patients. These data suggest that zebrafish *setd5* heterozygous mutants may be a valid model for drug screening and to study the molecular mechanisms of action underlying the effects of specific compounds in *setd5* haploinsufficiency conditions.

Considering the described activity of *Setd5* in controlling gene transcription, we focused on a comparative expression analysis of selected markers for neuronal pathways and synaptic components. Synaptic structure and functionality are the bases of neuronal communication and are critical for normal brain development and functionality, influencing behavior, learning and memory [38]. Although we observed a positive correspondence between the expression of markers of different neurotransmitter pathways and *setd5*, indicating that *setd5* haploinsufficiency affects neuronal signaling, a more consistent effect was found on the expression of genes involved in synaptic structure. Indeed, we found that heterozygous *setd5* adult brains show reduced levels of mRNAs encoding for Homer1b, a postsynaptic scaffold protein involved in synaptic plasticity [39] and for Dyrk1aa, a kinase involved in several cellular processes, including synapse function [40]. Moreover, a significant positive correspondence was also observed between the expression of *setd5* and Synaptophysin A, a protein of the presynaptic compartment, and *shank3b*, encoding a synaptic scaffolding protein that interacts with Homer [41]. No effect was observed in the expression of the postsynaptic scaffolding protein PSD95, indicating a specificity of action for *setd5*. Overall, these data suggest that *Setd5* may profoundly influence synaptic structure and function. In keeping with the social phenotype of *setd5* mutants, *dyrk1aa* [35] and *shank3b* [33] knockout zebrafish, as well as Synapsin knockout mice [42] display altered social behaviors, while *Homer 1* knockout mice show behavioral abnormalities related to ASD and Schizophrenia [43]. It is interesting to note that many of the risk genes that have been linked to ID and ASD disorders encode synaptic scaffolding proteins and changes in the expression of any of these proteins significantly affects synaptic strength or number, as well as neuronal connectivity in the brain [44]. The reduced expression of pre- and post-synaptic markers found in both zebrafish and mouse *Setd5* mutants [15,16], together with a decrease in the dendritic spine number observed in *Setd5*+/− mice suggests that brain hypoconnectivity could represent the main alteration caused by *setd5* haploinsufficiency that is responsible for the social impairments described. Perspective molecular studies will analyze the possible conservation in zebrafish of *SETD5*-dependent transcription fidelity during elongation [15]. Indeed, we cannot exclude a possible alteration of splicing processes in zebrafish *setd5* mutants.

## 4. Materials and Methods

### 4.1. Zebrafish Care

Zebrafish adults were housed in tanks at a constant temperature of 28 °C on a 14 h light/10 h dark cycle. Zebrafish embryos were obtained by natural mating and maintained at 28 °C in E3 zebrafish medium as previously described [45].

### 4.2. Generation of setd5 Mutant Zebrafish

For the CRISPR/Cas9 gene editing experiment, each zebrafish 1-cell zygote was injected in the cell with 2 nL of solution containing ~12.5 ng/µL of gRNA (20 nucleotide sequence complementary to the target: GGGAGACACGAATTCGGCAA), ~300 ng/µL of Cas9 mRNA [46] and 0.5% of Phenol Red as a tracer. Successful gene editing in mosaic embryos at 2 days post-fertilization (dpf) was confirmed by Melting analysis, performed using SensiFAST™ HRM Kit (Bioline) according to the manufacturer’s instructions in the Corbett Rotor-Gene 6000 machine (Qiagen, Hilden, Germany). To identify the individuals capable of transmitting *setd5* gene mutations through their germline, F0 generation adults were outcrossed with wild type individuals and the eventual heterozygous condition of F1 progeny was screened by Melting analysis, followed by mutation identification by Sanger sequencing. Presumptive off-targets were analyzed using Cas-OFFinder (CRISPR Rgen Tool, http://www.rgenome.net/cas-offinder/ (accessed on 26 July 2020)) and the modification of genes including a similar RNA guide target up to 4 mismatches was excluded by Sanger sequencing. Primers used are listed in Tables S2–S4.

### 4.3. Genotyping

Genomic DNA extracted from both larvae and adult fins was amplified by PCR using Bioline BIOTAQ™ DNA Polymerase kit and BIORAD iCycler thermocycler, according to the manufacturer’s instructions. PCR products were purified using NucleoSpin^®^ Gel kit and PCR Clean-up kit (Macherey-Nagel, Düren, Germany) and the concentration of total DNA was determined by NanoDrop™. Sequence data were obtained by Sanger method (GATC Biotech, Ebersberg, Germany) and analyzed with different tools, such as Tide software (https://tide-calculator.nki.nl/ (accessed on 26 March 2021)) [47], CRISP-ID software (http://crispid.gbiomed.kuleuven.be/ (accessed on 26 March 2021)) [48] and Poly Peak Parser software (http://yosttools.genetics.utah.edu/PolyPeakParser/ (accessed on 26 March 2021)) [49] to identify sequence alterations in *setd5* mutants. Primers used are listed in Tables S3 and S4.

### 4.4. Morphological Analysis

Images of zebrafish embryos at 48 hpf were obtained by the stereomicroscope Nikon SMZ1500n with a digital camera CoolSNAP-cf. Adult male fishes (10-months old) of F1 generation were anesthetized, weighted and then photographed by a camera. Eye area and body length were calculated by ImageJ software (RRID:SCR_003070).

### 4.5. Adult Brain Dissection

Each adult zebrafish male (10–12 months of age) was sacrificed, kept on ice, photographed to measure body length, weighed and then placed under a stereomicroscope to dissect the brain. The head was isolated by cutting with a sterile scalpel at the level of the anterior fins. Soft tissues were removed from the ventral side of the skull and eyes with surgical forceps. The skull was then opened, and the brain was transferred into TRIzol^®^ reagent (Invitrogen, Waltham MA, USA) or quickly photographed to perform brain measurements and then fixed for 6 h in 4% paraformaldehyde (PFA) at 4 °C, successively cryoprotected in 30% sucrose in phosphate buffer saline (PBS) overnight (O/N), sectioned using a cryostat (12 micron-thick sections) and finally collected onto polarized slides (SuperFrost^®^ Plus; MenzelGläser, Braunschweig, Germany).

### 4.6. Extraction of Total RNA and RT-qPCR

Total RNA was extracted and purified using RNAeasy Plus Mini (Qiagen) according to the manufacturer’s instructions. The concentration and purity of total RNA was determined by NanoDrop™. First strand cDNA was synthesized using QuantiTect Reverse Transcription Kit (Qiagen) according to the manufacturer’s instructions.

RNA expression levels were evaluated by quantitative reverse transcription-polymerase chain reaction (RT-qPCR) using the SYBR Green method (SensiMix SYBR kit; Meridian, London, UK), following the manufacturer’s protocol. Real time PCR and relative quantification of each gene expression was performed essentially as previously described [50]. Primers used are listed in Table S1. The transcript level of examined genes was normalized to *b-actin1* mRNA level according to standard procedures.

### 4.7. In Situ Hybridization on Frozen Tissue Sections

Brain samples of 10-months old WT adult male brains were fixed with 4% PFA in PBS for 6 h at 4 °C, then sectioned coronally (12 μm-thick) and collected on polarized slides. In situ hybridization on frozen tissue sections was performed as previously described [51], with some modifications. The *setd5* probe was diluted in hybridization buffer at 50 ng/mL and denatured at 85 °C. Cryosections were thawed and washed in PBS and incubated with the *setd5* probe at 65 °C O/N. Then, the slides were washed at 65 °C in a solution containing SSC 1X, 50% formamide, 0.1% Tween 20, while the final washing step were conducted in MABT (MAB, 0.1% Tween 20) at RT. After 1 h-long equilibration in blocking solution including MABT, 2% blocking solution (Roche, Basilea, Switzerland) and 20% lamb serum at room temperature (RT), slides were then incubated with anti-DIG Fab fragment conjugated with alkaline phosphatase (Roche, diluted 1:2500 in blocking solution) in a wet chamber at 4 °C O/N. After brief incubation in a buffer to inhibit endogenous alkaline phosphatase (100 mM Tris-HCL pH 9.5, 50 mM MgCl2, 100 mM NaCl, 2 mM levamisole and 0.1% Tween 20), slides were stained in BM purple staining solution (Roche) and placed in the dark at RT. The reaction was stopped by washes in PBST and samples were mounted with Aqua-Poly/Mount. After the staining procedure, images were acquired using stereomicroscope Nikon SMZ1500.

### 4.8. Immunofluorescence and Quantitative Analysis

Immunofluorescence analysis was performed on 24 hpf embryos. In brief, embryos were fixed in 4% PFA for 1 h at RT, cryoprotected in 30% sucrose in phosphate buffer saline (PBS) O/N, successively sectioned using a cryostat (12 µm-thick sections) and collected onto polarized slides. Sections have been washed and successively incubated O/N at 4 °C in a mixture containing both a rabbit polyclonal anti-pHH3 (1:400, cat. No. SC-8656-R, Santa Cruz Biotechnology, Dallas TX, USA) and a mouse monoclonal anti-HuC/D (1:100, cat. No. 16A11; ThermoFisher, Waltham MA, USA) primary antibodies previously used in zebrafish [52,53], diluted in 0.3% Triton X-100 (Merck, Darmstadt, Germany) in PBS. After washes, sections were incubated for 4 h at RT in a solution containing both Oregon Green 488 anti-rabbit (cat. No. 011038; Molecular Probes, Eugene OR, USA) and Alexa Fluor 594 anti-mouse (cat. No. A11032; Invitrogen) secondary antibodies, both diluted 1:500 in 0.3% Triton X-100 (Merck) in PBS. Successively, sections were washed and counterstained for 10 min at RT with 3 µg/mL Hoechst 33258 (Sigma-Aldrich) and finally slides were mounted with Aqua-Polymount (Polysciences Incorporated, Warrington, PA, USA). Adult brains’ cryosections (12 µm-thick) were washed, subject only to Hoechst 33258 counterstaining and mounted with Aqua-Polymount (Polysciences Incorporated). Fluorescence images of sectioned 24 hpf embryonic and adult brains were acquired with the microscope Nikon Eclipse Ti at 40× magnification, connected to the digital camera Nikon DS-Ri2, and equipped with the software NIS-Elements AR 5.11.03 (Nikon Corporation, Tokyo, Japan). Measurements were performed on CNS, excluding eyes and ventricles, by the ImageJ software.

### 4.9. Behavioral Analyses

All the behavioral tests were carried out in a quiet room, with a temperature between 27 and 28 °C, using system water. Adult male fishes (around 1-year age) and larvae at 6 dpf were recorded using a high-speed infrared camera (set to 30 frames per second) using Point Grey Fly Cap 2 software [54] or Noldus Media Recorder (Noldus, Wageningen, the Netherlands). All behavioral tests were conducted between 10 a.m. and 5 p.m. Behavioral recordings started after an acclimation period (1 h) to habituate fishes to a new environment.

For the open-field test, 6 dpf larvae were placed, one by one, into a 100 mm Petri dish and allowed to habituate for 1 min and then recorded for 5 min. The open field apparatus for adults consisted of a standard clear plastic fish tank that measured 20 × 22 × 37.5 cm, filled with 10 L of system water. Adult zebrafish were allowed to freely swim inside the tank, and videos were recorded for 5 min without habituating the fish to this setup. Video recordings were analyzed by Ethovision software (Noldus; RRID:SCR_000441) to calculate the total distance swum, the speed and the time spent moving or not moving.

The shoaling test was performed in a standard clear plastic fish tank that measured 20 × 22 × 37.5 cm filled with 10 L system water. A group of 4 fishes was placed in the novel tank and recorded for 10 min without adaptation. Video recordings were analyzed by Zebralab software (ViewPoint, Civrieux, France) to calculate the mean speed of the shoal, shoal polarization, Inter-Individual Distance (IID) and Nearest Neighbor Distance (NND) [31,32].

The social preference test [26] consisted of a plexiglass tank divided into 5 cells. The cells were separated by a transparent divider and water exchange was ensured by the presence of small holes. In the first round, the tested fish was placed into the center of the tank and recorded for 5 min in the presence of one male WT stimulus. In the second round, a new WT male stimulus was additionally introduced, and the experimental fish was recorded again for 5 min. The time spent the different tank zones was quantified using the video tracking software Ethovision software (Noldus; RRID:SCR_000441).

The setup of the social interaction test consisted of a mating tank, to separate the tested fish from the stimulus by a transparent divider. In the first round, the tested fish was placed into the left side of the tank and recorded for 5 min. In the second round, a WT male stimulus was introduced on the right side of the tank and the two fishes were recorded for 5 min. The distances between the nose-point of subjects were quantified by Ethovision software (Noldus; RRID:SCR_000441), using Multiple Body Point Module and the Social Interaction Module.

The social preference test and social interaction test were repeated after treatment with risperidone (Merck). At the onset of the experiment, the stock solution in dimethyl sulfoxide DMSO was diluted in system water to the final concentration of 170 μg/L [26]. Fishes were exposed to risperidone for 15 min prior to behavioral analysis.

### 4.10. Statistical Analysis

After verification of the normal distribution of the data, statistical analysis was performed with one-way analysis of variance (ANOVA) followed by the appropriate post hoc test or Student’s *t*-test, using the software GraphPad PRISM version 6.0 (RRID:SCR_002798). The same software was used to conduct linear regression analysis for gene expression studies. Value of *p* < 0.05 was considered significant.

## 5. Conclusions

Our study underlines the evolutionary conservation of *SETD5* activity providing further evidence of its role in regulating molecular, morphological and behavioral aspects underlying the *SETD5* haploinsufficiency phenotype. This work also highlights the effects of *Setd5* haploinsufficiency on other ASD-risk genes in zebrafish, suggesting potential convergent molecular mechanisms that may be commonly dysregulated in different groups of ASD patients. The heterozygous *setd5* mutant that we generated displays changes in social behaviors that are endophenotypes for autism and dysregulated expression of genes encoding crucial synaptic proteins, therefore representing a new model for *SETD5* haploinsufficiency. The rescue of the altered social interactions by risperidone is a promising indication of the suitability of this model for drug screening aimed at reversing more specifically the behavioral phenotypes, thus contributing to the development of future therapeutic treatments.

## Data Availability

The datasets used and/or analyzed during the current study are available from the corresponding author on reasonable request.

## Supplementary Materials

The following supporting information can be downloaded at: https://www.mdpi.com/article/10.3390/ijms24010167/s1.
