# Supplementary material for: CRISPR/Cas9-Induced Inactivation of the Autism-Risk Gene setd5 Leads to Social Impairments in Zebrafish
Source: Int J Mol Sci. 2022 Dec 22;24(1):167. doi: 10.3390/ijms24010167 (PMC9820161; doi:10.3390/ijms24010167)
Supplement: Supplementary file 1 [file ijms-24-00167-s001.zip › ijms-1900747-SI.pdf]

SUPPLEMENTARY MATERIALS

Table S1. Primers used in RT-qPCR or PCR

**Primers**	**Forward (5'-3')**	**Reverse (5'-3')**
setd5	GCATAGTGCTGTTCCCCCTT	CAGGGGCGTTGGTTTTTGTT
setd5 WT	CACCACTCCCAAAGCCAAGA	GGTCCGTCCATTGCCGAATT
setd5 MUT	CACCACTCCCAAAGCCAAGA	CATACTGGTCCGTCCATTCG
b-actin1	CGAGCAGGAGATGGGAACC	CAACGGAAACGCTCATTGC
elavl3	AGACAAGATCACAGGCCAGAGCTT	TGGTCTGCAGTTTGAGACCGTTGA
homer1b-202	TCCAGAGCCGCTTCATTCAG	CTGCTCCCCCATGTTGCTG
sypa	GCAAATCAGTTGGTTGCCCA	TTCTTGCACTCCACGCTCAT
sypb	CGTTCAGACTGCACCAGGTAT	CAGAAAGAGTCGCTCGGGTT
gad2	AAGCAGAAGGGATACGTGCC	CCGGTGTTTCCGGGACATTA
gad1a	CCATACAGTGTGGCCGTCAT	CATTTGTGTGCTGTGGCTCC
gad1b	ATATTCCACCGAGTCTGCGT	TGCCGCACTCCATCATCATT
th1	GACGGAAGATGATCGGAGACA	CCGCCATGTTCCGATTTCT
th2	CTCCAGAAGAGAATGCCACATG	ACGTTCACTCTCCAGCTGAGTG
slc6a3/dat	CGTCACCAACGGTGGAATCTA	TGCCGATGGCCTCAATTAGTA
dbh	TGCAACCAGTCCACAGCGCA	GCTGTCCGCTCGCACCTCTG
ddc	CAAAGGAGGTGGGGTCATCC	CACCGATGAGTGTGCCTGAT
serta	ACAACCGATGGAACACTCCC	CAACACCTGCCGGACATAAA
sertb	AGGAGACCAGCGTATGGGTA	GGGATTGTAGCTGGACAGGG
tph1a	CTGCCTGAGGAAAGCGAGAT	CATACATCAGCACGCGGTTC
tph1b	CTAAGAGCATACGGGGCTGG	GGACGCTGGATTGTCTTTGC
tph2	GGGCTGTGCAAACAAGATGG	CTCCTGGTAGCACGTGGTTT
vmat2	TGGAGCTCTGCAGCTTTTTGTGC	AACGCCGGCTCCAGCATAGC
hdc	TTCATGCGTCCTCTCCTGC	CCCCAGGCATGATGATGTTC
setd2	TGGATGCTCTGTTGAATCCTGA	AGACCTTCTGTCGACCCTGT
mll5	AGCCTTCAAGCCACCTGTAG	ACCTTCCCCCTGTACTCGAT
shank3a	GGCACTTATTACGCTGCTGGATCTG	CATGACGGCAAGCCTGGTGAAT
shank3b	CGGCCGTGGCAACAACAC	TTAAGCACATCGGTCAGGCTTTGT
mecp2	ACGTCTACCTTATCAACCCAGA	CCTTCCACGTCCAGAGGG
nrxn1a	GATGCAGTGCTGGTTAGGGT	ATGTCATCGGTGCCCACATT

nrxn1b	GGGGGACTACCTCAAACTGC	TCAATAACCGGCAGGTCGTC
PSD95/dlg4	CACCTGTCTTCTCTCTGCCA	ATGGCCGCCAGGTTCTTATC
syn1	CGCAGCTTAACAAATCCCAGT	ATGGTCTCAGCCTTGGCTTC
nsd1a-201	CAAGGGCTATCCGGTGGTTC	TTCGGACATCAAGAGCCCTG
nsd1b-201	TCAAAGGCTCACTCATCCCTG	TATTTCACTGGCGGTAGAGC
nsd1b-202	TCACAAAATGAAAGAGCCACCA	CGCTTGGGTATCTGCAAAGC

Table S2. Primers used in Melting analysis

Primers	Forward (5’-3’)	Reverse (5’-3’)
setd5	ACTCTGCGTCAGAGACCAGT	GGAGGGTCTGTACATCAGCG

Table S3. Primers used to generate PCR amplicons to be sequenced by the Sanger method

Primers	Forward (5’-3’)	Reverse (5’-3’)
setd5	GCAATGATGCCTTTTGTACACCAT	TGTTCTGTTTGTCTTTTGTTGCTAT
Off target_1	AGGGCTGCCTGAAAAACTTCT	CCCTTCCCCTTTTTCTGAAAGTC
Off target_2	AGCAACGGGTTTACTTGCTT	CTCTGGGAGCTGTCAGTGTG
Off target_3	CAGCCTCTTCTGTTATCAATTAGGT	AGGGGATCCTTGATGGGTGA
Off target_4	TGGCAAAGGTTATACAGTGGGAA	GACAGTGGAAAATGCAACGTCA

Table S4. Sequencing primers for PCR amplicons

Primers		Sequences (5’-3’)
setd5	**Rv**	TCTGTTTGTCTTTTGTTGC
Off target_1	**Rv**	AGATAGACTGGGCGATTTG
Off target_2	**Fw**	AGCAACGGGTTTACTTGC
Off target_3	**Fw**	CTTTGGATGCCAACAATG
Off target_4	**Fw**	TGCCATTCATCCTTGCAG

SUPPLEMENTARY FIGURES

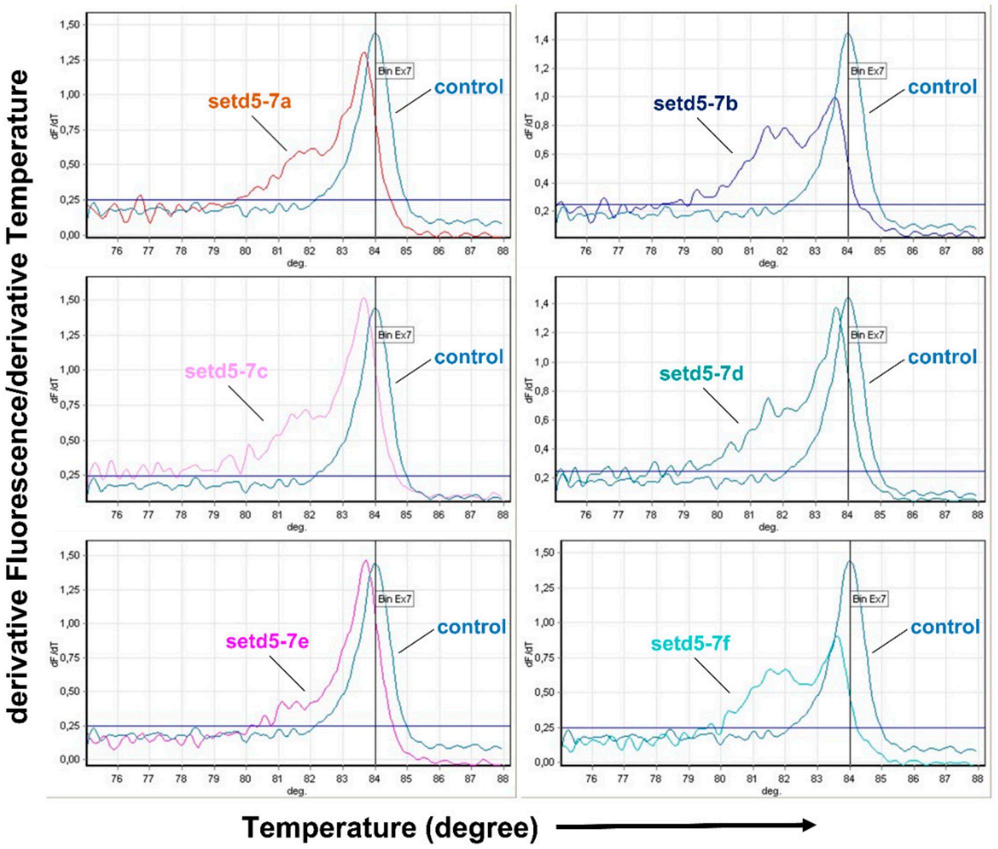

Figure S1: Efficiency of CRISPR/Cas9 gene editing system targeting *setd5* in zebrafish. Melting analysis of 6 representative zebrafish embryos injected with gRNA targeting a sequence encoding the Exon 7 of *setd5* gene along with zebrafish codon optimized mRNA encoding Cas9 endonuclease. Uninjected wild type *setd5* embryos were used as control.

(a)

WILD TYPE GATGAGAACACAACCGAAGGGTGGGAGACACGAATTCGGCAATGGA

Δ8 allele GATGAGAACACAACCGAAGGGTGGGAGACACGAA - - - - - - - - -TGGA

(b)

Δ8 allele translation

```
gatgagaacacaaccgaagggtgggagacacgaatggacggaccagtatga
 D  E  N  T  T  E  G  W  E  T  R  M  D  G  P  V  -
```

Figure S2: Sequence of frameshift mutation in *setd5* allele transmitted by CRISPR/Cas9-injected zebrafish founder. (a) Partial sequence of Exon 7 of *setd5* gene, including the guide RNA sequence (in green), the PAM sequence (in red), aligned with the sequence of mutated *setd5* gene (Δ8 *setd5*) lacking the 8 nucleotides underlined in the wild type sequence. **(b)** Putative appearance of a premature stop codon during translation of Δ8 *setd5* allele.

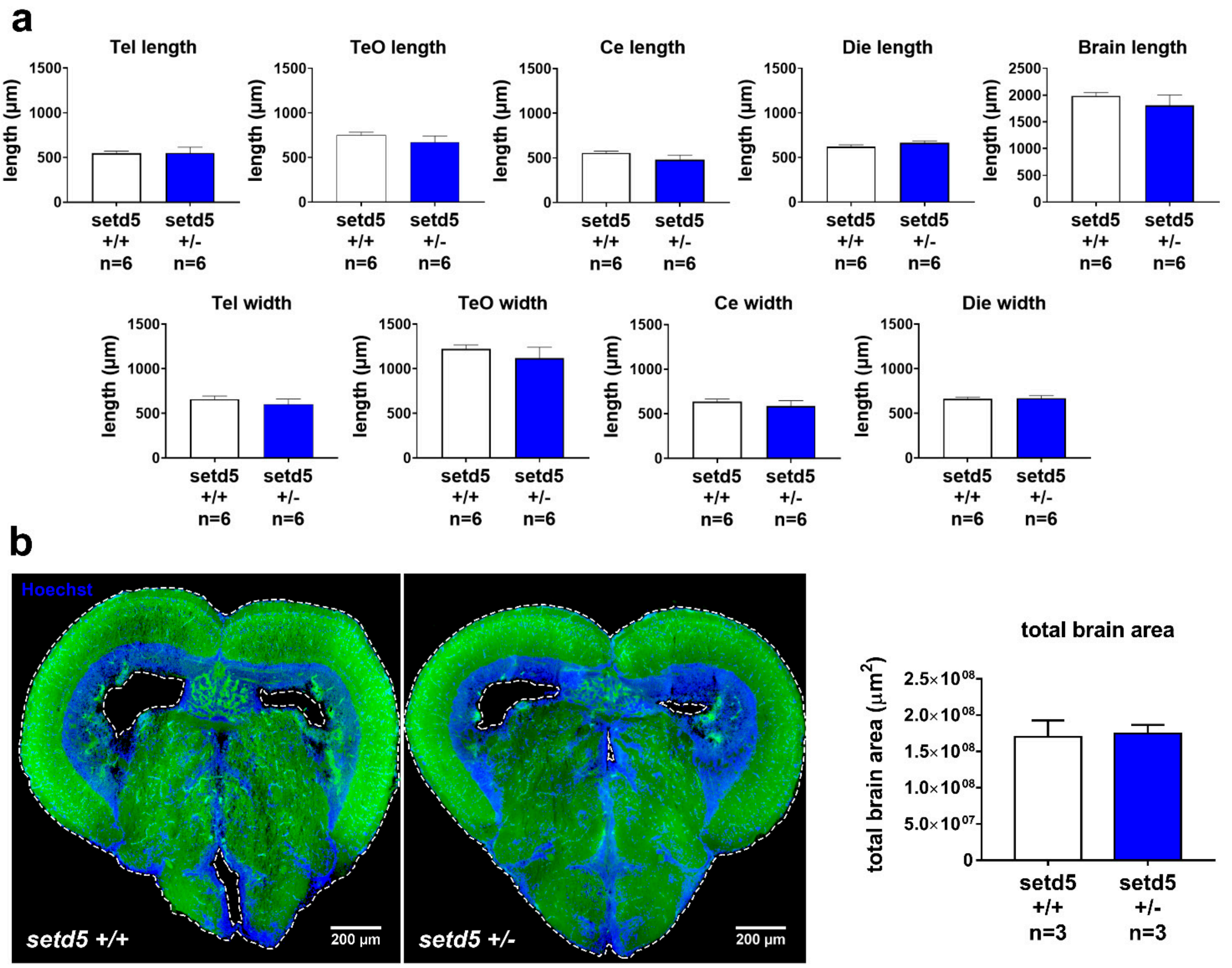

Figure S3: Morphometrical analysis of brain regions from mutant setd5 zebrafish adults. (**a**) Absolute Telencephalon (Tel) length and width, Optic tectum (TeO) length and width, Cerebellum (Ce) length and width, Diencephalon (Die) length and width and total brain length from 10-month-old *setd5*+/+ and *setd5*+/- zebrafish adults. (**b**) Representative images and quantification of central nervous system area from coronal sections from 10-month-old *setd5*+/+ and *setd5*+/- adult zebrafish brains. Hoechst counter-staining in blue. Sections 12 µm-thick. 40X magnification. (**a, b**) n=number of adult brains analyzed. Data are expressed as mean ± SEM. Statistical analysis was performed using (**a**) Student t-test or (**b**) Mann-Whitney test.

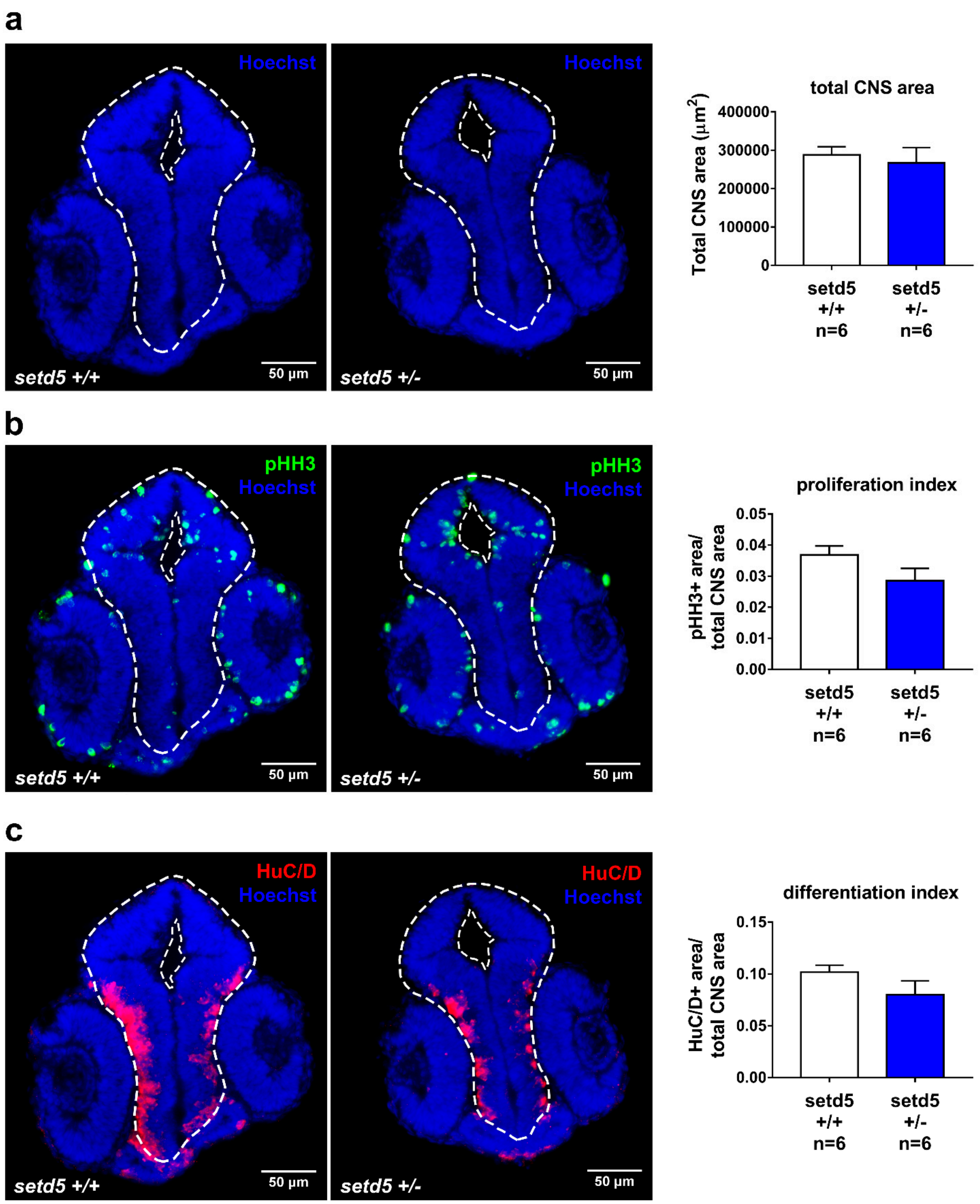

Figure S4: Evaluation of proliferation and differentiation index in developing central nervous system of mutant setd5 embryos. (**a-c**) Representative images and quantification of (**a**) developing central nervous system (CNS) area, immunofluorescence for (**b**) proliferation marker histone H3 phosphorylated in Ser-10 (pHH3) in green and (**c**) neural differentiation marker HuC/D in red on

coronal sections of CNS of 24 hpf embryos. (**a-c**) Hoechst counter-staining in blue was used to obtain total CNS area. Sections 12 µm-thick. 40X magnification. n=number of embryos analyzed. Data are expressed as mean ± SEM. Statistical analysis was performed using Student's *t*-test.

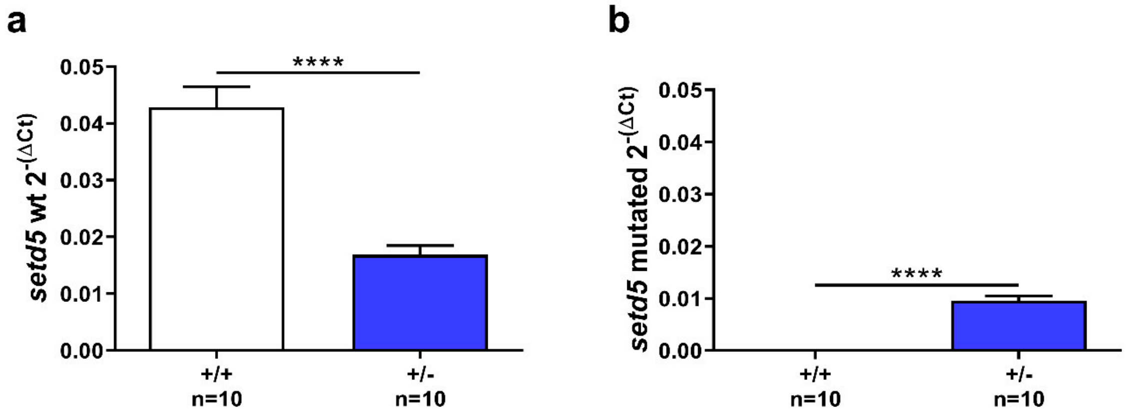

Figure S5: Expression of setd5 in mutant setd5 adult zebrafish brains. (a,b) Expression of wild type *setd5* (**a**) and the mutant *setd5* (**b**) in *setd5*+/+ and *setd5*+/- adult zebrafish brains, obtained by RT- qPCR analysis. The values are expressed as $2^{-(\Delta Ct)}$, using *b-actin1* as the housekeeping gene. n=number of adult brains analyzed. Data are expressed as mean ± SEM. Statistical analysis was performed using Student's *t*-test. ****p < 0.0001.

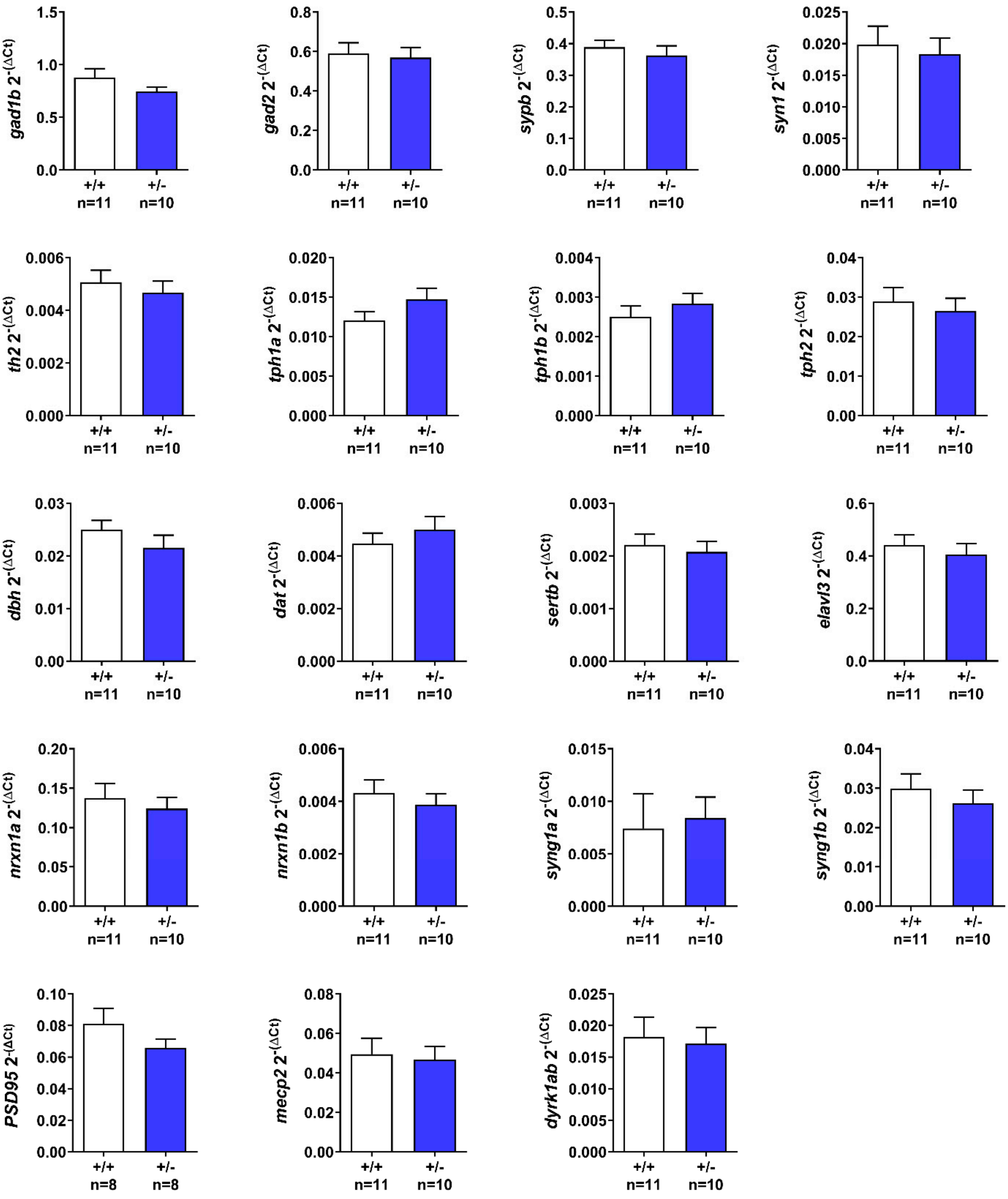

Figure S6: Expression of different genes encoding for regulators of different neurotransmitter pathways in mutant setd5 adult zebrafish brains. Expression of different genes encoding for regulators of different neurotransmitter pathways and proteins involved in neuronal activity in *setd5*+/+ and *setd5*+/- adult zebrafish brains, obtained by RT- qPCR analysis. The values are

expressed as $2^{-(\Delta Ct)}$, using *b-actin1* as the housekeeping gene. n=number of adult brains analyzed. Data are expressed as mean ± SEM. Statistical analysis was performed using Student's *t*-test test.
